# Phase Equilibrium and Interdiffusion in Poly(Vinyl Methyl Ether)-Water System

**DOI:** 10.3390/polym12112445

**Published:** 2020-10-22

**Authors:** Uliana V. Nikulova, Anatoly E. Chalykh

**Affiliations:** Frumkin Institute of Physical Chemistry and Electrochemistry Russian Academy of Sciences (IPCE RAS), 119071 Moscow, Russia; chalykh@mail.ru

**Keywords:** poly(vinyl methyl ether), phase behavior, water solution, interdiffusion, sorption, phase dissolution kinetic

## Abstract

The phase state diagram of the poly(vinyl methyl ether)-water system in a wide concentration range was obtained by the optical interferometry method. It was shown that this system was characterized by a complicated phase equilibrium with two lower critical solution temperatures, one of which was located in the concentrated region at 21 °C, and the other one in the region of a dilute solution at 31 °C. In the framework of the Flory–Huggins theory, pair interaction parameters were calculated for different parts of the binodal curves, and an attempt was made to reverse simulate the diagram in different conditions. It was suggested that the unusual character of the diagram was associated with the formation of a complicated complex between PVME and water in the middle region of the compositions. Concentration profiles for different temperatures were constructed. For the first time for this system, the numerical values of the diffusion coefficients of poly(vinyl methyl ether) (PVME) into water and water in PVME were obtained. Concentration and temperature dependences of diffusion coefficients were constructed and analyzed. The kinetics of water sorption in PVME was plotted, the clustering integral was calculated, and the approximate number of molecules in a water cluster was estimated. It was shown that in the dilute solution region upon passing through the binodal curve, the interphase disappeared immediately, and the remaining fluctuation of the concentration decreased in size with time. The kinetics of this process was estimated from the change in the size of such a particle.

## 1. Introduction

The study of phase equilibria and translational mobility of the components of polymers aqueous solutions is currently a promising and important direction in the field of physical chemistry of macromolecular compounds [1,2]. First of all, this is associated with the creation of biodegradable polymeric materials, in particular, packaging, insulating films, tapes, casings, coatings, etc. [3,4]. Modern medicine increasingly relies on effective and targeted treatment using biocompatible transdermal polymer systems supplied with ingredients, including drugs, that regulate their phase state in the human body [5]. Thus, technologies for the synthesis and application of intelligent materials based on hydrogels that are sensitive to temperature and allow regulating blood flow in blood vessels are actively developed [6,7]. It is obvious that aqueous solutions of such polymers should have a lower critical solution temperature (LCST) located in the area of the human body’s vital activity 20–36 °C. These conditions are met by poly(vinyl methyl ether) (PVME) and its copolymers.

Currently, aqueous solutions of PVME are traditionally used in these studies as model systems. Numerous works [8,9,10,11,12,13,14,15,16,17,18,19,20,21,22,23,24,25,26,27,28,29,30] show that the PVME-water system is characterized by a specific phase state diagram: two LCST coexist simultaneously in a binary system at the temperature-concentration field of the diagram. One of them is located in the concentrated solution area and the other one in the area of a dilute solution. Tanaka [8] called this type of diagram as a double-well-shaped phase diagram.

Initially, most of the studies were aimed at obtaining and reproducing data on the phase equilibrium of both binary [8,9,10,11,12,13,14,15,17,18,19,20,21,22,23,24,25,26,27,28,29] and ternary [16,30] systems. The methods of cloud-point curves [8,9,10,14,20], DSC (differential scanning calometry) [11,12,16,18,22,23,24,29] (including MTDSK—modulated temperature differential scanning calorimetry [19]), phase contrast microscopy [8], optical microscopy [15,29], X-ray scattering [15], dielectric spectroscopy [18], SALLS (small-angle light scattering) [20], SANS (small-angle neutron scattering) [21], and High-resolution ultrasonic resonator [22] were used earlier. Authors investigated the influence of factors, such as pressure [20], the introduction of salts [19], and solvents [13,16,21,29], on the phase equilibrium of this system. It is shown that with an increase in pressure from 0.1 to 800 MPa, the phase state diagram undergoes significant changes both in the region of concentrated and dilute solutions. The introduction of salts (NaCl, for example) shifts the binodal curve to the region of lower temperatures, and the LCST in the dilute solution region degenerates.

To identify the reasons for the phase decomposition of an aqueous solution of PVME with increasing temperature, studies have been carried out using Fourier-transform infrared spectroscopy (FTIR) [13,15,17,26,29] and Raman spectroscopy [27]. The most interesting works (Maeda [13], Zang [15], Zeng [17], Liu [26]) are those the authors of which analyzed in detail the evolution of the characteristic wavelengths from 1000 to 1100 cm^−1^ (blue shift for ν(C-0)) and from 3000 to 2800 cm^−1^ (red shift for ν(C-H)) during water absorption by PVME. Based on these data, Liu [26] identified three regions: in the range up to 40% PVME, it is easy to completely destroy the PVME·*n*H_2_O complex; range from 60 to 100% PVME, where the complex is stable in low temperature, and an intermediate region (from 40 to 60% PVME), where the complex is unstable. It is shown [2,13,15,26,28] that the “hydrophilicity” of the ether oxygen atom provides, on the one hand, the formation of hydrogen bonds with the hydrogen atoms of water. On the other hand, the presence of a hydrophobic methyl group leads to the effect of repulsion of water molecules and the formation of clusters that prevent the hydrophobic interaction of monomer units. It is assumed that the number of water molecules participating in the formation of the complex varies in the range from 2 to 8. The hydration number of oxygen of the ether group does not exceed two water molecules, and the hydrophobic complex can contain up to 5–7 water molecules. Increasing the temperature destroys the PVME·*n*H_2_O complex and leads to phase separation of the system.

Much attention is paid to phase equilibrium simulation [11,12]. It is assumed that two LCSTs are a consequence of the bimodal distribution of the PVME molecular weight, and each critical point is related to the corresponding fraction of the polymer. Molecular dynamics of aqueous solutions of hydrophilic polymers are studied only theoretically by estimating the lifetime of hydrogen bonds and their correlation function [9,10]. It is shown that the mobility of water molecules is reduced due to the structuring of water around hydrophobic groups. However, data on local diffusion coefficients in the PVME-water system are not obtained.

Despite numerous experimental and theoretical investigations, a number of fundamentally important questions have remained open. First, it is not clear what, from the thermodynamic point of view, is responsible for the presence of two critical temperatures. The previously stated assumption that this is a consequence of the bimodal distribution of PVME macromolecules is not confirmed by studies for monodisperse fractions of polyether [13,15,17,26,29]. Secondly, of interest is the difference between the phase separation temperatures of dilute and concentrated solutions. The first one is realized in the temperature range of 30–35 °C, the second at temperatures of 20–24 °C. Under these conditions, hydrogen bonds, according to FTIR data, “work”, and therefore the destruction of the complex cannot be the only cause of LCST. Third, the kinetic regularities of components mixing, the features of structure formation in the process of systems phase separation have not been analyzed experimentally before, and the processes of mutual and self-diffusion in PVME solutions have not been studied.

This article presented new experimental data on the phase equilibrium of the PVME-water system in a wide range of temperatures and concentrations. The thermodynamic parameters of mixing were also calculated, on the basis of which we would consider, in a new way, the features of the interaction of the components. For the first time, the coefficients of translational diffusion of PVME and water were obtained and generalized, as well as the results of simulation binodal curves in the framework of the quasi-chemical theory of solutions [1,31].

## 2. Materials and Methods 

Poly (vinyl methyl ether) (PVME) and distilled water were used as the objects of the research. The original PVME (Basf, Ludwigshafen, Germany) was a 50% aqueous solution: *M*_n_ = 45,000 g/mol, *M*_w_/*M*_n_ = 1.73, *T*_g_ = −31 °C. The refractive index of water is *n*_25_ = 1.332.

PVME films were formed from its 50% aqueous solution by casting onto the polyethylene terephthalate substrate at room temperature. After two days of drying at room temperature and 40% humidity, the films were annealed at 328 K in a thermo-cabinet XF050 (Moscow, Russia) and then for 4 h in a vacuum (VUP-5, Moscow, Russia) at a pressure of 10-5 mmHg. The remaining water content of the samples prepared in this way did not exceed 1.5 wt.%, according to thermal gravimetric analysis data (Netzsch TG209F1 Iris, Selb, Germany).

*Optical interferometry* [32]. The method was based on the principle of in situ registration of optical density distribution in the area of the conjugation of polymer and water and recording its change in time under isobaric-isothermal conditions of the process. The measurement method consisted of fixation of a PVME sample of 3 mm × 10 mm in size and about 120 μm thick between the diffusion cell glasses, the inner surfaces of which were covered with a layer of translucent metal (Ni+Cr) with a high reflection index. Using special fixtures, which were ~5% thinner than the PVME film, the sample was put into optical contact with the surface of the plates. While a small wedge angle θ ≤ 5° was established between the glasses. The polymer sample was placed in a flat capillary of the diffusion cell so that the interference strips oriented parallel to the edge of the wedge were to the longest side of the PVME sample. After assembly, the cell was thermoregulated at a set temperature for at least 30 min. Then, the capillary was filled with water.

All measurements were carried out on an ODA-2 IPCE diffusiometer (Moscow, Russia) [33] in the temperature range from 20 to 35 °C. The experiment was carried out in the heating-cooling mode with a step of 1 °C and thermostatting at each stage for at least 10–15 min. Special attention was paid to the kinetics of the phase particles’ formation and dissolution. For this purpose, a targeted photographic survey of the phase decay zones at different temperatures was carried out.

A helium-neon laser (λ = 632.8 nm) was used as a light source. Methods of processing of the interferograms, interdiffusion zones, and phase diagrams construction did not differ from those described earlier [34,35].

*Refractometry*. Information about the temperature dependences of the refractive indices of polymers was of fundamental importance in interpreting the optical density distribution profiles in the interdiffusion zone. In the range from 20 to 60 °C, a large difference was observed in the values of the PVME and water refractive indices (Δ*n* is about 0.137). An increment of the refractive index per interference strip equaled to ~0.022, providing a sufficiently high sensitivity in identifying changes in the composition in the phase conjugation zone. The refractometry data of the studied samples were presented earlier [35].

*Sorption measurements* [32]. The experiments on the sorption of water vapor by the polymer were carried out by the desiccator method at 25 ± 1 °C. The polymer was preliminarily kept in a desiccator with calcined CaCl_2_ granules at 0% humidity until a constant weight was established, then they were evacuated at a residual pressure of 10^−5^ mmHg. We measured the kinetics of changes in the mass of samples placed in desiccators with different vapor activity. Dried samples with a thickness of about 100 µm were placed in desiccators with different humidity levels (*p*/*p*_s_ 0.2, 0.4, 0.49, 0.67, 0.9, and 0.97). At regular intervals, the weight measurements of each sample were made on HR-200 A&D Co. LTD scales (Tokyo, Japan). The measurements were carried out until the sorption equilibrium was established, which, in the kinetic dependencies, was determined by the constancy of the sample mass over a period of 2–3 times longer than the time it was reached.

## 3. Results and Discussion

### 3.1. Conjugate Phase Zones

**Figure 1:**
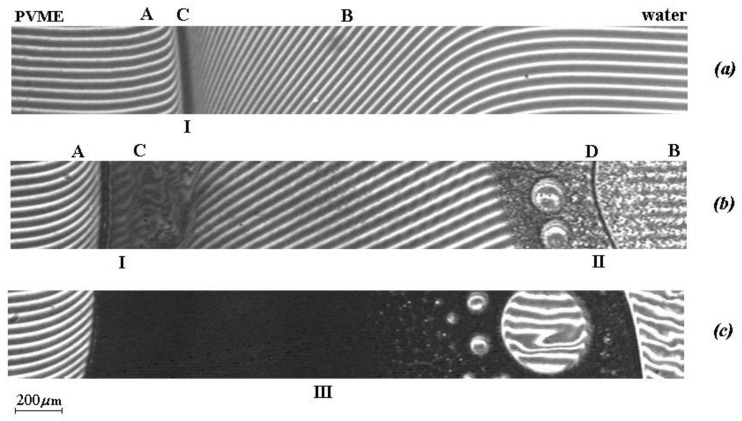
Interferograms of the interdiffusion zones of the poly(vinyl methyl ether) (PVME)-water system, obtained in the mode of a stepwise rise of temperature at (**a**) 22 °C, (**b**) 22 °C → 32 °C, (**c**) 32 °C → 35 °C (see text for details). The interaction time of the components with a stepwise rise of temperature corresponds to (**a**) 85 min, (**b**) 370 min, and (**c**) 397 min from the beginning of the experiment.

Optical interferometry allowed us to observe in situ the process of interdiffusion and phase decay at different temperatures in a wide range of compositions. Typical interferograms of diffusion zones obtained in the mode of a stepwise rise of temperature are shown in Figure 1. At *T* < 21 ± 0.5 °C, PVME is completely soluble in water. The interdiffusion zone at these temperatures corresponds to the region of smooth and continuous change in composition during the transition from PVME to water. As the temperature rises to *T* = 21 °C, the system undergoes phase decomposition in the region of the concentrated solution (I in Figure 1a). At this temperature, the interdiffusion zone is a superposition of three regions: region **A**—the dissolution of water in PVME, region **B**—the dissolution of PVME in water, region **C**—phase decay, within which are concentrated (localized) dispersions of PVME in water corresponding to the compositions of the coexisting phases of this area of the phase state diagram. With a further temperature increase to *T* = 31 ± 0.5 °C, the system undergoes a second phase decomposition in the region of dilute solutions of PVME in water (Figure 1b). The interdiffusion zone, in this case, is a superposition of five regions: region **A** – the dissolution of water in PVME, region **B**—the dissolution of PVME in water, region of phase decomposition in the zone of concentrated (**C**) and dilute solutions (**D**), and also the intermediate region of solubility between them. Finally, at *T* = 34 ± 0.5 °C, zones I and II merge into a single heterophase region III (Figure 1c), and the system as a whole takes on a classical form characteristic of systems with amorphous separation [1,31].

The analysis showed that even for a pseudo variant binary system in the presence of one variable parameter (temperature), the simultaneous existence of four phases is impossible. Thus, either the PVME-water system is not two-component (for example, due to the presence of complicated complexes between the components), or the influence of factors other than temperature and constant pressure should be taken into account, and then the system is a projection of a more complex volumetric diagram.

**Figure 2:**
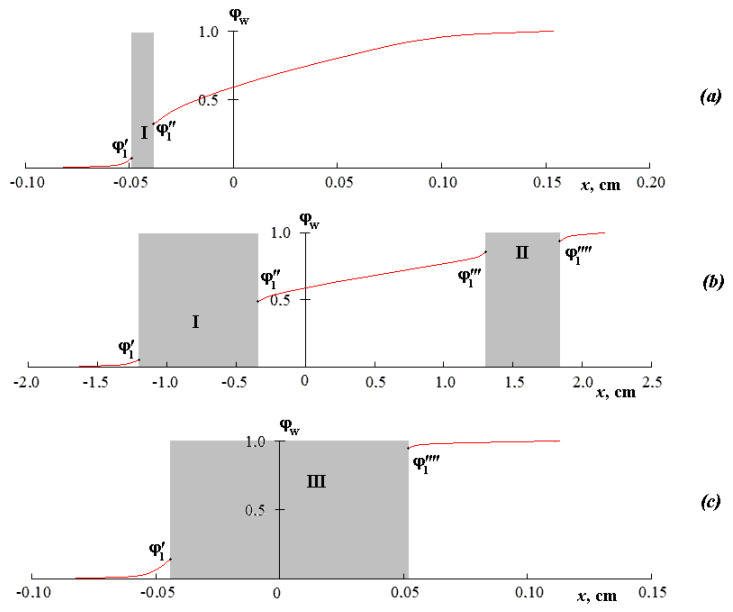
Concentration profiles of mixing PVME with water, obtained in the mode of the stepwise rise of temperature at (**a**) 22 °C, (**b**) 22 °C →32 °C, (**c**) 32 °C → 35 °C (see text for details). The interaction time of the components with a stepwise rise of temperature corresponds to (**a**) 85 min, (**b**) 370 min, and (**c**) 397 min from the beginning of the experiment.

Figure 2 shows the concentration profiles of the compositions’ distribution in accordance with the above interferograms. The gray areas of the concentration profiles correspond to the zones of the system phase decomposition, on both sides of which the compositions of the coexisting phases (ϕ_1_′, ϕ_1_′′, ϕ_1_′′′, ϕ_1_′′′′) are established. It is shown that ϕ_1_′, ϕ_1_′′, ϕ_1_′′′, ϕ_1_′′′′ under isothermal conditions are constant in time, but they change with temperature. Attention should be paid to the reproducibility of the values ϕ_1_′, ϕ_1_′′, ϕ_1_′′′, ϕ_1_′′′′ in the modes of increasing and decreasing the temperature, which allows us to speak of the equilibrium of the obtained values of the coexisting phases.

It is clearly seen that the concentration profile for the mutual solubility regions is characterized by smooth curves (to the left and to the right from I in Figure 2a). In the zone I of phase decay, a sharp change in concentration can be observed. For a temperature of 32 °C, the concentration profile consists of three sections of smooth curves corresponding to a concentrated solution, an intermediate region, and a diluted solution, and also two sections of a sharp change in concentration for the zones of phase decomposition I and II. For a temperature of 35 °C, the concentration profile of such a system has a classical form for a phase-separated system.

### 3.2. Phase State Diagram

**Figure 3:**
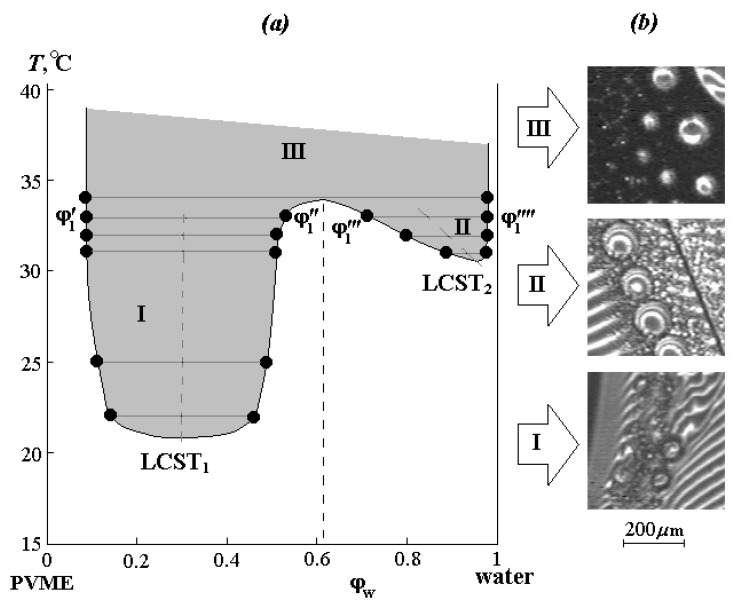
(**a**) PVME-water system phase state diagrams and (**b**) microphotographs of the phase structure for different zones (see text for details).

The phase diagram of the PVME-water system was constructed from the compositions of the coexisting phases (Figure 3a). It is characterized by the presence of two LCSTs (LCST_1_ and LCST_2_) and is in good agreement with the literature data [8]. Alekseev’s straight-line diameter rule was found, according to which the midpoints of the LCST_1_ and LCST_2_ regions form straight lines (dashed lines in Figure 3) passing through the minimums of the delamination regions boundaries. It can be seen that in the concentrated solution region, LCST_1_ is localized at 21 ± 0.5 °C and a water concentration of 0.32 vol.%, and in the diluted solution region, LCST_2_ corresponds to 31 ± 0.5 °C and a water concentration of about 0.95 vol.%. Upon reaching 34 °C at a water concentration of approximately ~0.6 vol.%, regions I and II merge, forming a single zone III of phase decomposition. It should be noted that the phase reversal region corresponds to the Alekseev diameter for the studied systems.

Figure 3b also shows microphotographs illustrating the phase structure of dispersions formed along isotherms in different zones of the phase diagram. If in the zone I, these are small formations from 10 to 50 μm, which retain their size for a long time, in zone II, the dispersed phase of PVME first precipitates in the form of small particles ~10–20 μm, then the particle size rapidly increases to 400 μm, and inside these formations, a secondary phase decay occurs. Zone III is characterized by an abundance of fine phases up to 20 μm. We assume that the presence of large phase particles in this region is associated with the kinetics of their formation at the initial stage of phase decomposition.

In accordance with the classical Flory–Huggins theory [1,31], using the previously developed method [35], we calculated pair interaction parameters χ for different regions of the phase diagram using the equation:

(1) $$\chi =\frac{\frac{ln\left(\varphi_{1}^{’’}/\varphi_{1}^{’}\right)}{r_{1}}-\frac{ln\left(\varphi_{2}^{’’}/\varphi_{2}^{’}\right)}{r_{2}}}{2\left(\varphi_{2}^{’}-\varphi_{2}^{’’}\right)}$$

where $\varphi_{1}$ and $\varphi_{2}$ are volume concentrations of the first and second components, respectively, $r_{1}$and $r_{2}$ are their polymerization degrees, *‘* and *‘‘* refer to different coexisting phases, χ is the Flory–Huggins interaction parameter assuming the absence of its concentration dependence and is the average value for the pair interaction parameters $\chi_{12}=\chi +\frac{\partial \chi }{\partial \varphi_{1}}\varphi_{1}$ and $\chi_{21}=\chi +\frac{\partial \chi }{\partial \varphi_{2}}\varphi_{2}$.

**Figure 4:**
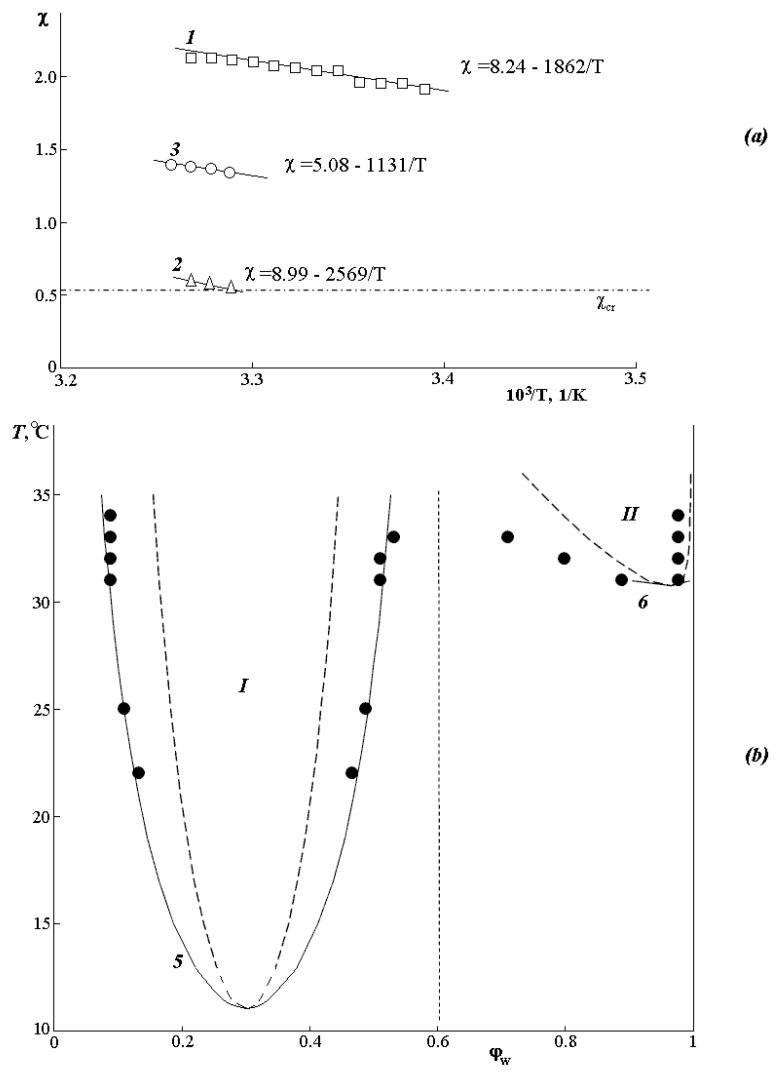
(**a**) Temperature dependence of χ, according to phase separation in I (1), II (2), and III (3) zones and (**b**) simulation of the phase state diagram. The dash-dotted line (4) shows the calculated value of χ_cr_, according to the Flory–Huggins theory. 5—phase equilibrium simulation for the PVME-PVME·*n*H_2_O system according to the χ− 1/T dependence for zone I, 6—phase equilibrium simulation for the PVME-water system according to the χ− 1/T dependence for zone II. Black points are experimental data of phase equilibrium.

Figure 4a shows that the temperature dependences of the Flory–Huggins parameter are linear. Dashed line 4 shows the calculated value $\chi_{cr}=\frac{1}{2}{\left(\frac{1}{\sqrt{r_{1}}}+\frac{1}{\sqrt{r_{2}}}\right)}^{2}$ for the PVME-water system. The expansion of the parameter χ into the entropy (first term of the polynomial) and enthalpy (second term of the polynomial) components (χ_S_ and χ_H_, respectively) shows that the contributions of χ_H_ are close to each other, while χ_S_ for all three bands is significantly different. Based on this, we assume that the thermodynamics of mixing of components in different zones is entropic in nature.

It can be seen that the values of the pair interaction parameter for zone II are close to χ_cr_, and the critical temperature, estimated from the point of intersection of the dependence χ− 1/T, coincides with the experimentally found value. The calculated values of LCST for zones I and III, estimated by the same method, differ significantly from the experimental ones. To explain this fact, the values of the pair interaction parameters χ_12_ and χ_21_ were determined. It was found that the values of the pair interaction parameters χ_12_ and χ_21_ in zones I and III differ significantly from each other, while for zone II, they completely coincide. This suggests that zones I and III are characterized by a concentration dependence of the Flory–Huggins parameter associated with the low thermal stability of the PVME·*n*H_2_O complex.

Nevertheless, based on the obtained equations for χ− 1/T, we tried to simulate fragments of the phase state diagram (Figure 4b) according to the equations for binodal and spinodal curves [1,31]:

(2) lnφ1’+(1−r1r2)φ2’+r1χ(φ2’)2=lnφ1’’+(1−r1r2)φ2’’+r1χ(φ2’’)2lnφ2’+(1−r2r1)φ1’+r2χ(φ1’)2=lnφ2’’+(1−r2r1)φ1’’+r2χ(φ1’’)2

(3) $$\frac{1}{r_{1}\varphi_{1,s}}+\frac{1}{r_{2}\varphi_{2,s}}-2\chi =0$$

The analysis showed that for the diluted solution region, there is a good agreement with the experimental data for the PVME-water system; 6 in Figure 4b corresponds to binodal and spinodal curves. In the concentrated solution area (left side of the diagram), simulation in the framework of the same approach gave us a mixed result. Good agreement with the experimental data (curves 5 in Figure 4b) was obtained for the case of extrapolating the left side of the diagram to the entire concentration range under the assumption that one of the components is PVME, and the other is the PVME·*n*H_2_O complex with several water molecules from 2 to 6 hydrated for each PVME functional group. In this case, the experimental points (black circles) correlate well with the calculated binodal curve (curve 5). It should bear in mind that the calculated value of LCST_1_ is ~10 °C lower than that obtained experimentally.

Thus, the assessment of the phase state diagram and the thermodynamics of mixing of components in different temperature-concentration regions allowed us to assume that the phase equilibrium in the PVME-water system is due to the formation of the PVME·*n*H_2_O complex, and in general, the system should be considered not as a binary but as a three-component PVME-water–PVME·*n*H_2_O. Then, the left part of the diagram (to the left of the vertical dashed line) corresponds to the amorphous decomposition of the PVME-PVME·*n*H_2_O system, and the right side to the amorphous decomposition of PVME-water, when the PVME·*n*H_2_O complex is destroyed.

### 3.3. Sorption

**Figure 5:**
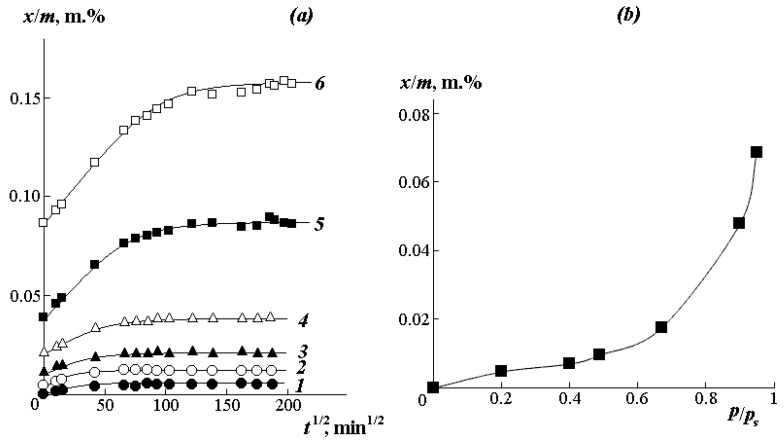
(**a**) Kinetic curves of interval sorption and (**b**) isotherms of water vapor sorption by PVME at 25 °C for vapor activity *p*/*p*_s_ 0.2 (1), 0.4 (2), 0.49 (3), 0.67 (4), 0.9 (5), and 0.95 (6).

Figure 5a shows the kinetic curves of interval sorption of water vapor at different humidity. It can be seen that the higher the vapor activity *p*/*p*_s_ under isothermal conditions, the larger the slope of the initial portion of the curve, and the greater the amount of water sorbed by the polymer. In accordance with Rogers’ classification [32], the kinetics of the establishment of an equilibrium state has a Fick character. It is known that in this case, the boundary concentrations of the sorbate quickly reach equilibrium values and remain unchanged during the entire process of establishing sorption equilibrium. Usually, such conditions are met when the rate of the structure rearrangement in the sorbent surface layer is higher than the rate of diffusion of sorbate molecules in the bulk of the polymer.

For determining the effective diffusion coefficients, kinetic curves were constructed in relative coordinates (x/m)(x/xmax)−t1/2, where x/m is the amount of water absorbed at each moment of time *t*; $x/x_{max}$ is equilibrium amount of sorbed water. The diffusion coefficient was determined from the initial rectilinear section of the integral curve in accordance with the formula [32]:

(4) $$D=\frac{tg^{2}\alpha \cdot l^{2}\cdot \pi }{16}$$

where *l* is a sample thickness in cm; *D* is a diffusion coefficient (cm^2^/s); *α* is the angle of inclination of the straight line according to the dependencies (x/m)(x/xmax)−t1/2.

The numerical values of the obtained effective diffusion coefficients are presented in Table 1.

Water vapor sorption isotherms were plotted from the equilibrium parts of the kinetic curves (Figure 5b). The isotherm has a weakly expressed S-shaped character and belongs to the IV type according to Rogers classification [36]. This type of isotherm is traditionally described in the framework of the “double sorption” model [37]:

(5) $$C=C_{FH}+C_{L}$$

The model assumes that some of the sorbed water molecules dissolve in PVME in accordance with the Flory–Huggins solutions theory (*C_FH_*). Another smaller part of the sorbate is immobilized on the accessible active sites of macromolecules monomer units, which is described by the Langmuir monolayer sorption equation (*C_L_*). Accordingly, the relationship between the relative water vapor pressure and the concentration of sorbate in PVME is described by the Flory–Huggins equation:

(6) $$ln\left(\frac{P}{P_{S}}\right)=ln\left(C_{FH}\right)+\left(1-\frac{1}{r}\right)\left(1-C_{FH}\right)+\chi {\left(1-C_{FH}\right)}^{2},$$

where $\frac{P}{P_{S}}$ is the relative vapor pressure, $C_{FH}$ is the bulk capacity of the sorbate in the sorbate + sorbent system, χ is the Flory–Huggins interaction parameter, r is the degree of polymerization of the polymer sorbent, and the Langmuir equation is as follows:

(7) $$C_{L}=\frac{C_{\infty }k\frac{P}{P_{S}}}{1+k\frac{P}{P_{S}}},$$

where $C_{\infty }$is the sorption capacity of active centers in PVME, k is the energy characteristic.

The constants of Equations (6) and (7) were determined by the numerical method of “gradient descent”, minimizing the average deviation of the calculated isotherm from the experimentally obtained one.

In the initial region of the isotherm up to *p*/*p_s_* < 0.3, where the Langmuir model is predominantly realized, water molecules fill free active centers, which are –O–CH_3_ groups located in vacancies of the excess free volume of the polymer. The share of such centers, estimated according to the ratio $C_{\infty }/C_{max}$, is 15 mass.%.

**Figure 6:**
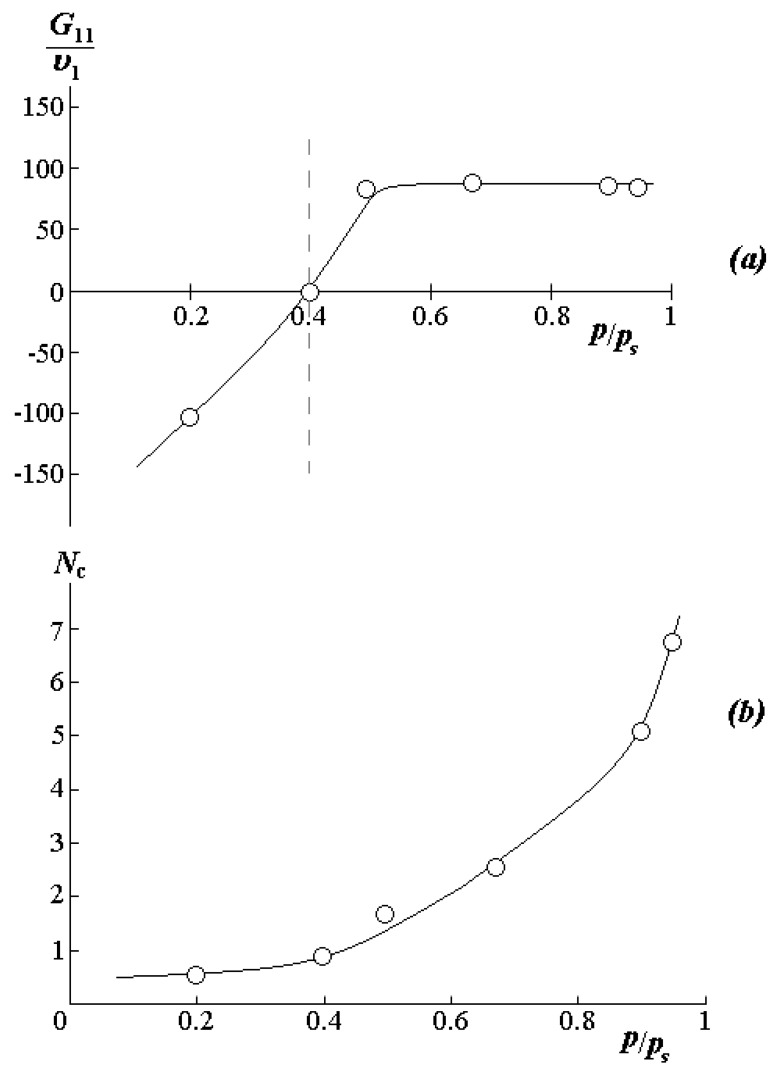
(**a**) Dependence of the clustering integral and (**b**) the average number of molecules in a cluster from the steam activity at 25 °C.

In the range of relative humidity from 0.35 to 1.0, the sorption isotherms are well approximated by the Flory–Huggins equation with pair interaction parameters χ varying in the range from 2 to 3. These data correlate well with the χ data in Figure 4a. Additional information on the state of water molecules in PVME was obtained using the Starkweather equations [38]. Recall that the quantitative characteristic of the formation of associate-clusters of water molecules in the sorbent matrix is the clustering integral $\frac{G_{11}}{\upsilon_{1}}$ (Figure 6a):

(8) $$\frac{G_{11}}{\upsilon_{1}}=-\left(1-\varphi_{1}\right)\left[\frac{\partial \left(\frac{a_{1}}{\varphi_{1}}\right)}{\partial a_{1}}\right]-1$$

where $\upsilon_{1}$, $\varphi_{1},a_{1}$ is the molar volume, volume composition, and activity of the sorbate, respectively. For an ideal solution, $\frac{G_{11}}{\upsilon_{1}}=-1$. For the systems studied by us, the border value of the sorption capacity characterizing the start of cluster formation, $\frac{P}{P_{S}}=0.4$ (marked with a dotted line in Figure 6). The average number of solvent molecules in a cluster can be obtained according to the Starkweather–Brown equation [38]:

(9) $$N_{c}=\varphi_{1}\left(\frac{G_{11}}{\upsilon_{1}}+1\right)+1$$

According to the sorption data at 25 °C and an increase in volume water concentration from 0.5 to 6.9 vol.%, $N_{c}$ rises from 0.5 to 6–7 (Figure 6b). In this case, the concentration of 6.9 vol.% actually corresponds to the binodal curve in the concentrated solution region. Thus, it can be assumed that the phase decomposition in the zone I is associated with a rapid increase in the number of water molecules in the cluster, which is formed around the PVME functional group. While the phase decay in zone II is caused by the decomposition of a similar cluster with an increase in temperature to 32 °C.

### 3.4. Particles Dissolution Kinetics of Dispersed Phases

**Figure 7:**
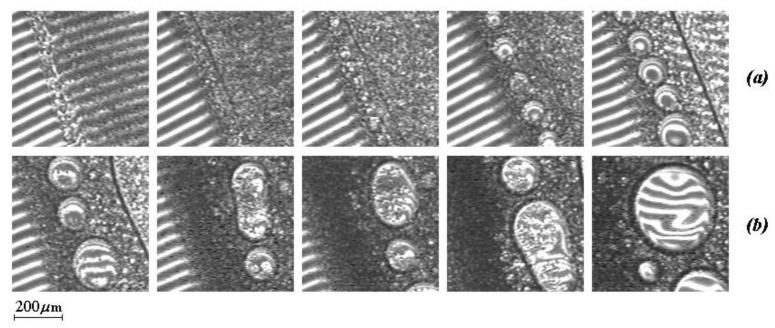
(**a**) Phase structure for decomposition zone II upon reaching 32 °C and after 109, 216, 344, and 564 s, and (**b**) phase structure for decomposition zone II when the temperature rises to 33 °C and after 81, 131, 288, and 806 s.

This study is of practical interest associated with the formation and subsequent destruction of functional phase particles, which were previously mentioned in [6,7]. The starting point of the model study was the formation of a gradient diffusion zone in the PVME-water system in the dilute region near the binodal curve. If, after the formation of the diffusion zone, the temperature is raised to 32 °C (above the LCST_2_), a phase decomposition occurs in a certain region of compositions (Figure 7). Within a few seconds (up to 100 s), dispersions of the aqueous phase in the PVME matrix and the PVME phase in water are formed. These dispersions are separated by a phase boundary corresponding to the position of the phase reversal region on the phase diagram in the zone II of phase decay.

Under isothermal conditions, the rapid growth of microphases from 10 to 100 μm is observed over time. Simultaneously, their coalescence occurs according to Smolukhovsky [39] with the formation of macroparticles ranging in size from 300 to 400 μm. The process of the Oswald ripening is also possible. Similar effects were observed earlier on oligomeric systems in [40,41].

**Figure 8:**
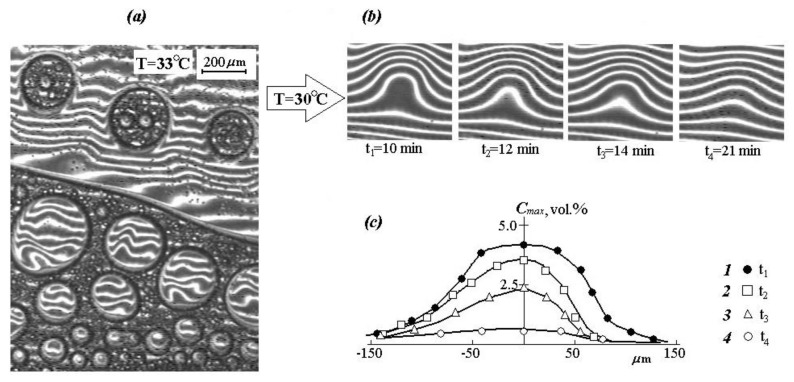
(**a**) Maturation of the PVME phases, (**b**) kinetics of phase dissolution upon transition through (**c**) the binodal curve and their amplitude: 1—*t*_1_ = 10 min; 2—*t*_2_ = 12 min; 3—*t*_3_ = 14 min; 4—*t*_4_ = 21 min.

It is interesting to note that with a repeated increase in temperature from 30 to 33 °C, a secondary phase decomposition is observed, which is clearly identified in macrophases (Figure 8a). This process is completely equilibrium and is reproduced in heating-cooling cycles.

To study the mechanism of the dissolution kinetics and diffusion of PVME macromolecules in aqueous solutions, the case of a sharp cooling of dispersions after the formation of disperse systems is of great importance. That is, the figurative point of the systems is outside the boundary curve.

It was found that at the first stage of the gradient structure evolution, the particle dissolution proceeds mainly due to local mass transfer in the interphase region. At the second stage, the interphase boundary transforms into a diffusion one, the dimensions of which are continuously changing. At the third stage, concentration profiles—vertical interference strips—reach the “particle” center. At this stage, the “particle” can be considered as a heterophase fluctuation [42], and the process of its dissolving can be considered in the framework of the classical diffusion theory of finite size “particle” dissolution (Figure 8b). From an experimental point of view, the kinetics of this stage of the process can be estimated from the change in the amplitude of the concentration profile (Figure 8c) using the equation:

(10) $$C_{max}=\frac{Q}{2\sqrt{\pi Dt}}$$

where *Q* is the amount of a substance in a nanoparticle (droplet), *D* is the diffusion coefficient, and *t* is time [43].

The diffusion coefficient of PVME in a diluted solution was determined using the ratio logC=log Const−12Dt. The numerical value of the calculated effective diffusion coefficient is then 2.5×10^−7^ cm^2^/s.

### 3.5. Diffusion

It has been established that the diffusion fronts motion in the region of concentrated and dilute solutions in coordinates $X-t^{1/2\;}$ under isobaric isothermal conditions of the process is linear. This indicates the diffusion mechanism of the spontaneous mixing of components in the region of true solutions (below the binodal curve). Here, *X* is the diffusion coordinate at the selected concentration, *t* is the observation time. The numerical values of the interdiffusion coefficients with a step equal to the concentration increment were calculated by the Matano–Boltzmann method [43,44].

**Figure 9:**
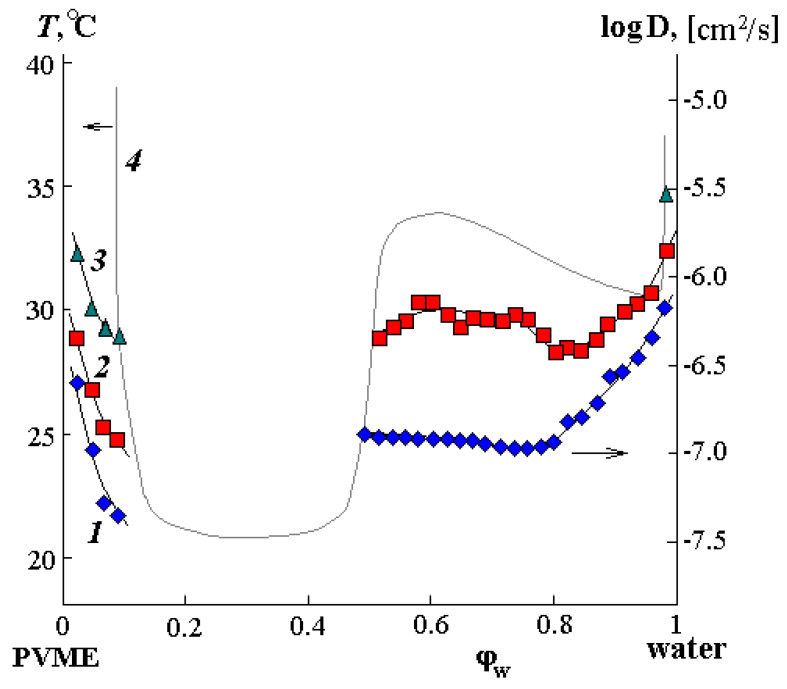
Concentration dependence of the interdiffusion coefficient for the PVME-water system at 25 °C (1), 30 °C (2), and 35 °C (3)—axis on the right. 4—phase state diagram (left axis).

Figure 9 shows the concentration dependences of the interdiffusion coefficients for temperatures of 25, 30, and 35 °C (curves 1, 2, and 3, respectively). For comparison, the figure shows a phase diagram (curve 4). It is clearly seen that for all temperatures in the concentrated solutions region (to the left of the binodal curve), a decrease in log*D* is observed as we approach the two-phase region boundary. It should be noted that the calculated values of the effective diffusion coefficient according to the sorption data completely coincide with curve 1, supplementing it at lower composition values. Such behavior of the interdiffusion coefficient is associated with the presence of a labile region inside the amorphous decomposition diagram, at the boundary of which the thermodynamic multiplier dlnadlnφ in the equation D=D*dlnadlnφ tends to zero [45].

In the dilute solutions region, diffusion has a more complex character since, in this case, the contribution of the thermodynamic correction to the value of the interdiffusion coefficient is determined by the distance of the figurative point from the binodal curve. The concentration region of mutual solubility at 35 °C is very small here (about 2 vol.% PVME) and log*D* = −5.55. This value is in good agreement with the self-diffusion coefficient of water [10]. For a temperature of 25 °C, the interdiffusion coefficient sharply decreases with an increase in the PVME content in the solution, but at medium values of the concentration (up to the binodal curve), it is monotonic. We assume that this is due to the presence of a complex, which hinders the translational mobility of molecules. The behavior of curve 2 in Figure 9 in the middle region of compositions is the most interesting: after the interdiffusion coefficient sharp decrease in the dilute solution region (similar to how it was at 25 °C), log*D* passes through a minimum and slightly increases. We associate this effect with the approach of the system to the second phase decay (zone II), from which it is separated by 1 °C. Consequently, it can be assumed that the existing complex begins to undergo changes (practically disintegrate), which, on the one hand, leads to an increase in the diffusion rate of components in this zone, but on the other hand, predetermines a rapid phase decomposition.

**Figure 10:**
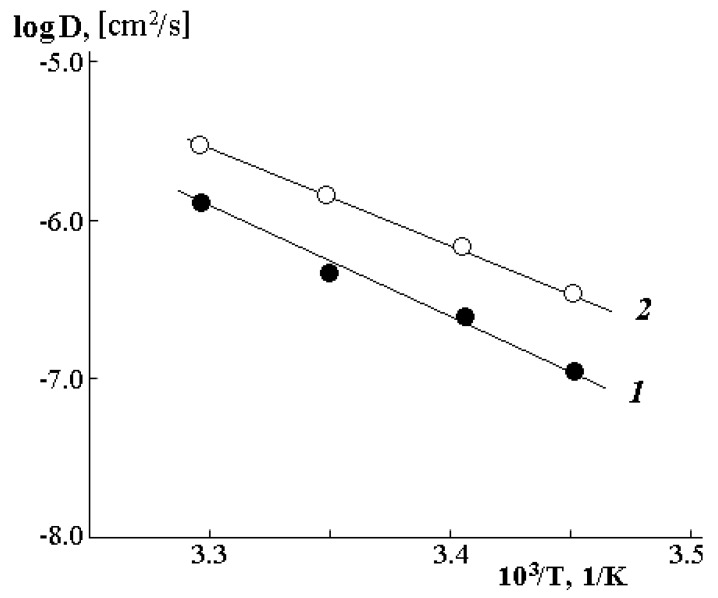
Temperature dependence of the interdiffusion coefficients into the PVME matrix (1) and into the water matrix (2).

The temperature dependence of the interdiffusion coefficients is plotted in the coordinates of the Arrhenius equation and is shown in Figure 10. It is clearly seen that in the investigated range of temperatures and compositions, it has a linear character both in the case of diffusion of water molecules into the PVME matrix (1 in Figure 10) and during diffusion of PVME into water (2 in Figure 10). The slope of such straight lines allowed us to calculate the diffusion activation energy, the numerical values of which correspond to 5.8–6.5 kJ/mol.

## 4. Conclusions

We have shown that the phase equilibrium in aqueous solutions of PVME is a complex projection of a multi-component system that coexists at different temperatures. Thus, in the temperature range below 31 °C, the phase diagram corresponds to amorphous separation in the PVME-PVME·*n*H_2_O system, while above 31 °C, the PVME·*n*H_2_O complex is destroyed, and the phase diagram corresponds to the binary PVME-water system. At the same time, the intermediate regions of mutual solubility are obviously associated with the inertial effects of dehydration of functional groups of PVME and the redistribution of unbound water molecules. The kinetics of the formation and dissolution of phase particles is consistent with the Oswald mechanism, and the phase size in the diluted solution region can reach 400 μm. In this case, a secondary phase decay is observed within such macrophases.

Sorption studies have shown that in the concentrated solution region, the number of water molecules in a cluster formed around the functional group of PVME sharply increases. According to our calculations, up to 6–7 water molecules are combined in such a cluster, which is in good agreement with the literature data.

For the first time for the PVME-water system, we obtained numerical values for the diffusion coefficient. The log*D* values (cm^2^/s) vary in the range from −7 to −5.5, with an increase in temperature from 25 to 35 °C. A good correlation is observed between the data obtained both from sorption measurements and from direct contact of polymers by the optical interferometry method. The analysis of the concentration dependences of the diffusion coefficients showed that the diffusion rate decreases sharply when approaching the binodal curve and changes monotonically in the middle region of the compositions. A noticeable effect on diffusion is exerted by the decomposition of the PVME·*n*H_2_O complex in the middle region of the compositions—the diffusion of the components here slightly increases. Based on the temperature dependences, the diffusion activation energy was estimated, the numerical values of which correspond to 5.8–6.5 kJ/mol.

## Figures and Tables

**Table 1:** Effective coefficients of diffusion *D* of water vapor by poly(vinyl methyl ether) PVME at 25 °C for different values of the vapor activity *p*/*p*_s_ and volume concentrations of water φ_w_

*p*/*p*_s_	φ_w__,_ vol.%	log*D*, cm^2^/s
0.2	0.5	−6.23
0.4	0.7	−6.44
0.49	1.0	−6.35
0.67	1.7	−6.70
0.90	4.8	−6.91
0.95	6.9	−7.10
